# The CANadian Pediatric Weight Management Registry (CANPWR): Study protocol

**DOI:** 10.1186/1471-2431-14-161

**Published:** 2014-06-23

**Authors:** Katherine M Morrison, Samah Damanhoury, Annick Buchholz, Jean-Pierre Chanoine, Marie Lambert, Mark S Tremblay, Glenn Berall, Jill Hamilton, Anne Marie Laberge, Laurent Legault, Lehana Thabane, Monica Jakymyshyn, Kathryn A Ambler, Geoff D C Ball

**Affiliations:** 1 Department of Pediatrics, McMaster Children’s Hospital, McMaster University, Hamilton, ON, Canada; 2 Population Health Research Institute, McMaster University, Hamilton, ON, Canada; 3 Stollery Children’s Hospital, Alberta Health Services, Edmonton, AB, Canada; 4 Department of Agricultural, Food and Nutritional Science, University of Alberta, Edmonton, AB, Canada; 5 Children’s Hospital of Eastern Ontario, Ottawa, ON, Canada; 6 Department of Pediatrics, BC Children’s Hospital; University of British Columbia, Vancouver, BC, Canada; 7 Department of Pediatrics, Université de Montréal, CHU Sainte Justine, Montreal, QC, Canada; 8 Department of Pediatrics, University of Ottawa, Ottawa, ON, Canada; 9 Department of Pediatrics, North York General Hospital, University of Toronto, Toronto, ON, Canada; 10 Department of Pediatrics, The Hospital for Sick Children, University of Toronto, Toronto, ON, Canada; 11 Department of Pediatrics, Montreal Children’s Hospital; McGill University, Montreal, QC, Canada; 12 Department of Pediatrics, University of Alberta, Edmonton, AB, Canada

**Keywords:** Pediatric, Obesity, Family, Treatment, Canada, Health services

## Abstract

**Background:**

Over recent decades, the prevalence of pediatric obesity has increased markedly in developed and developing countries, and the impact of obesity on health throughout the lifespan has led to urgent calls for action. Family-based weight management interventions that emphasize healthy lifestyle changes can lead to modest improvements in weight status of children with obesity. However, these interventions are generally short in duration, reported in the context of randomized controlled trials and there are few reports of outcomes of these treatment approaches in the clinical setting. Answering these questions is critical for improving the care of children with obesity accessing outpatient health services for weight management. In response, the CANadian Pediatric Weight management Registry (CANPWR) was designed with the following three primary aims:

1. Document changes in anthropometric, lifestyle, behavioural, and obesity-related co-morbidities in children enrolled in Canadian pediatric weight management programs over a three-year period;

2. Characterize the individual-, family-, and program-level determinants of change in anthropometric and obesity-related co-morbidities;

3. Examine the individual-, family-, and program-level determinants of program attrition.

**Methods/Design:**

This prospective cohort, multi-centre study will include children (2–17 years old; body mass index ≥85^th^ percentile) enrolled in one of eight Canadian pediatric weight management centres. We will recruit 1,600 study participants over a three-year period. Data collection will occur at presentation and 6-, 12-, 24-, and 36-months follow-up. The primary study outcomes are BMI z-score and change in BMI z-score over time. Secondary outcomes include anthropometric (e.g., height, waist circumference,), cardiometabolic (e.g., blood pressure, lipid profile, glycemia), lifestyle (e.g., dietary intake, physical activity, sedentary activity), and psychosocial (e.g., health-related quality of life) variables. Potential determinants of change and program attrition will include individual-, family-, and program-level variables.

**Discussion:**

This study will enable our interdisciplinary team of clinicians, researchers, and trainees to address foundational issues regarding the management of pediatric obesity in Canada. It will also serve as a harmonized, evidence-based registry and platform for conducting future intervention research, which will ultimately enhance the weight management care provided to children with obesity and their families.

## Background

Obesity has become a major public health issue in Canada. One in three Canadian children and youth are overweight or obese; a three-fold increase over the last three decades [1]. There is an increasing recognition of obesity-related health consequences during childhood into adulthood [2]. The development of obesity and its subsequent co-morbidities is influenced by a complex inter-relationship between factors that interact at multiple levels (e.g., physiology, individual activity, physical activity environment, food consumption, food production, individual psychology, and social psychology), which has been conceptualized as an obesity system map [3]. Examining and understanding how these factors relate to one another and change over time can help to tailor interventions to the unique needs of children with obesity and their families rather than a more traditional “one-size-fits-all” approach to weight management [3,4].

Lifestyle behaviour changes represent the foundation of pediatric weight management programs [5,6]. Several recent reviews have highlighted that comprehensive, family-based interventions are effective approaches for managing pediatric obesity [7-9]; however, intervention-mediated changes can vary substantially between individuals, and the causes of this variability remain understudied. Characterizing these variations and influences of potential determinants of obesity and obesity-related health outcomes at the individual-, family-, and program-level can help to guide the development of more effective interventions [10].

To improve weight management care, the individual factors that differentiate those boys and girls whose health outcomes improve *versus* those with no improvements or worsening health outcomes, as well as factors related to attrition and recidivism require additional study. Changes to lifestyle (nutrition, physical activity, sedentary activity) habits are usually promoted through individual and/or group-based counseling to encourage their adoption and maintenance [9,11]. The valuable outcomes of behavioral lifestyle interventions in the treatment of childhood obesity have been recently highlighted [8,12]. Limited research suggests that mental health issues predict poor response to the treatment of pediatric obesity [13]. Other factors predicting weight management program outcomes include participation in an exercise program at baseline [14], young age [15,16] with pre-pubertal children being more responsive than teens, and lower body mass index (BMI) z-score at baseline [17].

A number of family-level characteristics have been found to predict treatment outcomes in children with obesity [18]. A recent systematic review demonstrated that interventions that applied cognitive behavioral therapy for parents and children and the inclusion of rewards from parents were associated with improvements in children’s weight status. Furthermore, healthy diets among children were associated with high intakes of healthy foods and low intakes of unhealthy foods (e.g., fast foods) among family members, and less parental food restriction [19]. Although continued family attendance at clinical appointments [20,21] and high readiness to change lifestyle habits [22,23] are associated with health improvements in children with obesity, the factors that influence attendance and readiness remain poorly delineated.

At the program-level, limited research is available on the influence of program characteristics on obesity management and obesity-related health outcomes. Children and parents who complete a structured lifestyle and behavioural intervention achieve greater reductions in weight status than non-completers [8]. Intervention intensity (e.g., number of clinical contact hours) may be a key determinant of treatment efficacy, but the optimal number of contact hours is uncertain, and the nature of how and with whom these clinical hours are spent is unknown [8]. Our understanding of the effectiveness of program-related factors including intervention modality (e.g., group *vs* individual sessions), disciplinary approach (e.g., unidisciplinary *vs* multidisciplinary), and behavioural techniques (e.g., self-monitoring nutrition/physical activity habits, regular weighing, goal-setting) [24] in managing pediatric obesity remains incomplete. Multi-centre research will allow us to examine the influence of determinants of responsiveness to obesity management in diverse clinical environments that extend across cultures and contexts within the Canadian health care environment [25].

Despite evidence supporting the effectiveness of strategies to manage pediatric obesity [8], little evidence is available regarding the sustainability of changes in health outcomes achieved through behavioural interventions in pediatric obesity as highlighted in multiple systematic reviews [8,9] and reports [26]. A recent systematic review identified four trials that evaluated the maintenance of weight change after lifestyle and behavioural interventions and concluded that improvements in obesity status can be sustained over a 12-month follow-up period [8]. Children with obesity can lose and maintain weight loss up to five [15] and 10 years [27] following family-based lifestyle and behavioural interventions; however, these findings remain unique in the literature and fail to consider potential changes in other factors that are often targeted within interventions (e.g., nutrition, physical activity, cardiometabolic risk factors).

Related to sustainability of behavioural change and to program outcomes is the degree of attrition from clinical programs. Similar to attrition levels reported from adult obesity management programs, [28-30] dropping out of care is common in pediatric weight management programs, with reports suggesting 27–73% attrition [31]. Though not studied extensively, children’s age, mental health, and family socioeconomic status at presentation have been linked with attrition [30]. Descriptively, parents’ perception of the quality of the program at treatment onset is an important predictor of whether families will prematurely discontinue care [24].

With the aforementioned issues in mind, several questions remain unanswered; questions that are critical for improving the management of pediatric obesity. For instance: How do health outcomes beyond weight and BMI change during participation in weight management programs? What are the determinants of changes in body size and obesity-related co-morbidities? Are changes in obesity and obesity-related co-morbidities sustainable beyond 12 months in real-world, clinical settings? Are the determinants of change different for sustained *versus* transient changes in health outcomes? What individual, family, and contextual factors predict program attrition? To address these questions, the CANadian Pediatric Weight management Registry (CANPWR) study was designed with the following aims (Table 1):

**Table 1:** **Objectives**, **hypotheses**, **measures**, **and methods of analysis**

**Objective**	**Hypothesis**	**Outcome measure (C = Continuous; B = Binary)**	**Methods of analysis**
**Primary**	Change in BMI z-score will be influenced by child/youth, family, and program characteristics consistent with our theoretical model	BMI z-score (C)	Hierarchical/multilevel modeling
Document changes in anthropometric, lifestyle, behavioural, and obesity-related co-morbidities in children enrolled in Canadian pediatric weight management programs over a three-year period			
**Secondary**	Change in cardiometabolic health outcomes will be influenced by child/youth, family, and program characteristics consistent with our theoretical model	Systolic and diastolic blood pressure (C)	Hierarchical/multilevel modeling
1) Document changes in anthropometric, lifestyle, behavioural, and obesity-related co-morbidities in children enrolled in Canadian pediatric weight management programs over a three-year period;			
		Blood glucose (Fasting & 2 hr post glucose load) (C)	
		Total cholesterol/HDL-C ratio (C)	
		Triglyceride (C)	
		Fitness (C)	
		Quality of Life (C)	
		Lifestyle behaviours (C)	
2) Characterize the individual-, family-, and program-level determinants of change in anthropometric and obesity-related co-morbidities;	Individual-, family-, and program-level determinants will be identified that predict sustainability of change from years 1 – 3.	BMI z-score (C)	Hierarchical/multilevel modeling
3) Examine the individual-, family-, and program-level determinants of program attrition.	Individual-, family-, and program-level determinants will differentiate those who dropped out of the program	Drop out from the program between enrollment and 1 year (B)	Hierarchical/multilevel modeling
			Logistic regression
**Exploratory analyses**	We will identify interaction terms between some individual, family and program determinants	All outcomes	Hierarchical/multilevel modeling
Identify what works best for what groups of individuals or families			
**Sensitivity analyses**	As above	All outcomes	1) Analysis with multiple imputation
1) Imputation methods			
2) All outcomes analyzed simultaneously to account for correlation among them			2) MANCOVA
			3) GEE
3) Serial correlation of all outcomes over time			

*MANOVA*: multivariate analysis of covariance.

*GEE*: Generalized estimating equations.

1. Document changes in anthropometric, lifestyle, behavioural, and obesity-related co-morbidities in children enrolled in Canadian pediatric weight management programs over a three-year period;

2. Characterize the individual-, family-, and program-level determinants of change in anthropometric and obesity-related co-morbidities;

3. Examine the individual-, family-, and program-level determinants of program attrition.

The following hypotheses will be tested:

### Primary hypothesis

1. Change in children’s BMI z-score will be influenced by individual-, family-, and program-level determinants.

### Secondary hypotheses

1. Individual-, family-, and program-level determinants will influence changes in anthropometric, lifestyle behaviour and cardiometabolic health measures over a three-year period.

2. Individual-, family-, and program-level determinants will influence the sustainability of change in BMI z-score over a three-year period.

3. Individual-, family-, and program-level variables will predict program attrition.

## Methods/Design

### Study design

This prospective cohort, multi-centre study will include children (2–17 years old; BMI ≥85^th^ percentile) who consent to participate and are enrolled in one of eight participating weight management centres affiliated with children’s hospitals in Hamilton, ON (McMaster Children’s Hospital; coordinating site); Vancouver, BC (BC Children’s Hospital); Edmonton, AB (Stollery Children’s Hospital); Toronto, ON (The Hospital for Sick Children and North York General Hospital); Ottawa, ON (Children’s Hospital of Eastern Ontario); and Montreal, QC (Montreal Children’s Hospital and CHU Sainte Justine). The study centres will continue their current program [25], but data collection for outcomes and determinants will be standardized amongst centres. It should be noted that all of these programs are in urban centres, are in secondary or tertiary care environments, may have relatively wide geographic referral areas and are funded under a single payer system. The current manuscript has been written in accordance with the Strengthening the Reporting of Observational Studies in Epidemiology (STROBE) guidelines and checklist [32].

### Study population

All children and youth enrolling in a participating tertiary care weight management centre will be eligible to participate. Beginning in 2013, we will recruit 1,600 study participants over three years and will follow them for up to three years. Exclusion criteria are children younger than 2 years or older than 17 years, age- and sex-specific BMI < 85^th^ percentile and lack of fluency in spoken and written English or French.

### Ethics approval & confidentiality

Research ethics and administrative site approvals were received at all eight participating sites prior to study initiation. Collected information will remain confidential; individual names will not be used for any purpose. Each participant will be assigned a unique ID number, which will be used as the participant’s identifier. Records that identify participants will be kept confidential and are only available to the research team for contact purposes. All participant study binders will be stored in lockable filing cabinets at each site, which are under the supervision of the local site leads. Any data presented publically or published as a result of this research will ensure participant anonymity.

### Measurements

Health outcomes and purported determinants that are evidence-based and measured at each centre with methodologies that have been standardized and harmonized through the initial phase of CANPWR will be included. The primary outcome is change in BMI z-score (WHO definition). Secondary health outcomes include absolute BMI [33], waist circumference, hip circumference, cardiometabolic measures (blood pressure, glycemia (fasting and two-hour blood glucose), lipid profile (total cholesterol/high density lipoprotein cholesterol ratio, and triglyceride), liver enzymes (AST, ALT)), aerobic fitness and quality of life (Peds QL). The determinants to be evaluated for their influence on change of the primary and secondary outcomes include individual, family, and program characteristics. Characteristics of the *Individual* include demographic (e.g. age, sex, ethnicity, immigrant status), biological (e.g. BMI z-score at baseline, medical history including medication use and other health disorder(s)) and life style behaviours (e.g. dietary intake, family eating patterns, sedentary activity, sleep, readiness to change, nutrition and physical activity habits). *Family* characteristics include family health (e.g. parental weight status, family history of type 2 diabetes, coronary heart disease), socioeconomic status, family structure (e.g. number of people living in household), and readiness to change nutrition and physical activity behaviours at baseline. *Program* characteristics will include intervention modality (individual *vs* group-based care), parental participation, participant satisfaction with care, proportions of clinical time families spend with different disciplines, number of clinic hours attended, clinical status at time of study visit (active, inactive or discharged), and adherence to scheduled clinic visits (proportion of planned visits attended).

### Data collection

Information will be retrieved at the baseline visit, followed by 6-, 12-, 24-, and 36-months follow-up. Data collection will be done within the weight management centres, all of which are in ambulatory environments. A comprehensive questionnaire will be administered and includes information on demographics, child lifestyle behaviours, and readiness to change lifestyle habits. Many demographic and lifestyle-related questions were derived from the *Canadian Health Measures Survey*[34], which was done to enable comparisons between study participants and a nationally-representative sample of Canadians. Family eating patterns will be evaluated using a validated questionnaire [35] that has predicted changes in BMI z-score amongst children enrolled in a weight management program. To promote participant retention, site specific newsletters will be periodically mailed or emailed to families. This newsletter will serve as a reminder to participants of their upcoming appointment, will encourage families to contact CANPWR researchers if their contact information changes during the study and will update them on the study progress. If study participants stop attending the weight management program (either inactive or discharged), a research visit separate from clinical care will be arranged to enable data collection. When data are missing or if families discontinue study participation, whenever possible, data will be retrieved from children’s medical records to populate study-specific case report forms. Participating families will receive up to $100 in the form of gift cards as tokens of appreciation. All de-identified study data will be entered into a secure, encrypted *iDataFax* web-enabled software application for central storage at the Population Health Research Institute (PHRI) (McMaster University; Hamilton, ON). A complete list of the contributors to the CANPWR study including the Central Coordinating Site and the research staff at each site is listed in Additional file 1.

### Inter-site calibration procedures

Standardized, accurate, valid, reliable, and reproducible measures are fundamental to the success of CANPWR. Quality assurance and quality control procedures will be implemented, recorded, and monitored. Intra- and inter-site variation will be evaluated and reported as outlined below. CANPWR quality assurance processes have begun with harmonization and review of the methods applied at each site for collection of outcome variables and determinants. Furthermore, training in completion of data collection forms and monitoring of completion of questionnaires will occur at each site and update courses will be offered as required. For laboratory measures, participation in accepted international quality assurance programs (e.g., College of American Pathologists) will be assured and monitored. Quality control processes are the operational techniques and activities used to fulfill requirements for quality. For physical measures, duplicate measures will be taken at defined intervals at each site to evaluate duplicate measures both within and between staff. These will be centralized and monitored on a periodic basis. A central research coordinator will travel between sites to fulfill several purposes, including ensuring study binders are complete and secure, to review data completion procedures with the study teams, review the periodic duplicate measures recorded, review training and monitor data collection (assuring consistency obtained from chart) and evaluate consistency of measures between sites.

### Statistical analysis

The results of patient demographics and baseline outcome variables (both primary and secondary) will be summarized using descriptive summary measures, expressed as mean (standard deviation) or median (minimum-maximum) for continuous variables and number (percent) for categorical variables. We will use multi-level or hierarchical modeling to analyze the data to address the primary and secondary aims [36] to determine the relationship between outcomes and individual-, family-, and program-level characteristics (Table 1). We will report the results as the estimate of the measure of association—model coefficients for continuous outcomes, odds ratios (OR) for binary outcomes or hazard ratio (HR) for time-to-event outcomes, corresponding 95% confidence intervals, and associated p-values. We will report p-values to three decimal places with p-values less than 0.001 reported as p < 0.001. All statistical tests will be performed using two-sided tests at the 0.05 level of significance. The Bonferroni method will be used to adjust the level of significance for testing for secondary outcomes to keep the overall level at α = 0.05. We will assess colinearity using the variance inflation factor (VIF) which measures the extent to which the variance of the model coefficients will be inflated. We will consider variables with VIF > 10 colinear and we will exclude them from the analysis [37]. We will perform all analyses using Statistical Analysis System (*SAS*) *version 9.2* (Cary, NC, USA).

Sensitivity analyses will be performed to assess the robustness of the results. First, there is likely to be missing data that will likely increase with time. We will use multiple imputations to handle missing data [38]. Second, there is likely to be high inter-correlations among outcomes. We will use a multivariate analysis of covariance (MANCOVA) approach to analyze all outcomes simultaneously. This method accounts for possible correlations among all outcomes and provides for a global assessment of the impact of each predictor variable with an indication of where differences exist. Third, we will use generalized estimating equations (GEE) assuming an auto-regressive correlation structure to account for possible serial correlation of measurements within a patient overtime [39]. Unlike ordinary linear regression, GEE allows accounting for possible serial correlation of outcomes within a patient over time.

### Statistical power

Given a sample size of 1,600 and 41 variables or determinants to be included, the ratio of participants to variables is 39:1. Previous studies have shown that a fitted regression model is likely to be reliable and stable when the number of independent predictors is less than the total sample size divided by 20 [40]. Considering 40%, 25% or 15% dropout (n = 960, 1,200, 1,360 respectively), a two-sided test with significant level α = 0.05/4 = 0.0125 gives 90% power to detect a small R-square 0.0313 -0.0442 for 41 determinants. Thus, we have high power to test the effects of the described variables. For secondary hypothesis one (Table 1), we intend to examine three additional outcomes. To account for multiple hypothesis testing two-tailed α = 0.0125, we will continue to have power exceeding 90% to examine the influence of the determinants on the outcome at one year. For secondary hypothesis two, we intend to examine the determinants for sustainability of weight loss. To do so, we will examine the determinants for maintenance or decline of BMI z-score from year one to year two. If we conservatively estimate that at least 30% will have an increase in BMI z-score over that time period, we have 90% power to detect a variable that will increase this by 1.3 fold – and this is true even if we have 40% drop out from our study. Similarly, if the proportion of participants whose BMI z-score increases from year one to year two is higher, our power would be > 90%. Findings from several published trials suggest these assumptions are appropriate [21,41]. For secondary hypothesis three, we will examine the determinants of program drop out within one year of enrollment. We are sufficiently powered to detect an OR of 1.2 if 25% or more of those who commence a program drop out, and to detect an OR of 1.3 if only 15% drop out of the program.

### Pilot study

The foundational work for the protocol development occurred within the context of evaluation of a pilot study that was undertaken at five centres, supported by a grant from the Canadian Institutes of Health Research – CANNeCTIN program (http://www.cannectin.ca). We demonstrated feasibility of recruitment and conducting of this study within real world environments. Further, we agreed on a core data set of outcomes and measurement protocols, developed harmonized data collection, and the case report forms. These have been further modified based on completeness of data collection and data quality evaluations from the pilot study. The feasibility of multiple ethics approvals, financial and data transfer agreements, and translation of materials into both official languages were also verified.

## Discussion

### Contributions

The Canadian Clinical Practice Guidelines for the Management and Prevention of Obesity [6] highlighted and underscored the “mismatch between the high prevalence and significance of pediatric obesity and the limited knowledge base from which to inform treatment strategies” [6,9]. Therefore, CANPWR will construct the first harmonized, evidence-based registry and platform that identifies the key determinants of weight change in eight pediatric weight management centres across Canada. The registry will contain detailed information regarding individual-, family-, and program-level determinants of change in health outcomes and behaviours. It will make it possible to compare these determinants of change in a large, diverse population of children and their families throughout Canada. The outcomes of this study are expected to contribute important information on the sustainability of change in weight status and obesity-related co-morbidities. We expect to identify subgroups of children who do and do not respond well to treatment paradigms, which will inform how health services should be enhanced or modified to meet the needs of children with obesity and their families. By prospectively collecting data from a large number of families and by comparing the characteristics across centres, CANPWR will help us understand the determinants of program attrition.

### Limitations

We have chosen to address a limited population, as the minority of children with obesity will be managed at the secondary or tertiary care level. As lifestyle behaviours are currently collected by self-report at all of the centres we have relied on self-report for these variables, which may differ from objectively measured data. Further, we were challenged to find validated questionnaires that have demonstrated predictive capabilities within the childhood obesity treatment environment.

### Future plans

We intend to undertake this project at sites affiliated with academic institutions with the express purpose of extending the data collection methods to primary and secondary level care practices. Furthermore, we plan to incorporate additional measures in the future as consensus is reached among the clinical and academic communities on outcomes of greatest importance and as our team grows and develops to include investigators, collaborators, and trainees with complementary expertise in mental health and other relevant fields.

## Abbreviations

ALT: Alanine transaminase; AST: Aspartate transaminase; BMI: Body mass index; CANPWR: CANadian Pediatric Weight management Registry; HR: Hazard ratio; GEE: Generalized estimating equations; MANOVA: Multivariate analysis of variance; OR: Odds ratio; PHRI: Population Health Research Institute; SAS: Statistical Analysis System; STROBE: Strengthening the Reporting of Observational Studies in Epidemiology; VIF: Variance inflation factor; WHO: World Health Organization.

## Competing interests

The authors declare that they have no competing interests.

## Authors’ contributions

Contributed to study conception and design (KMM, GB, AB, JPC, JH, ML, LL, MST, GDCB, LT). Participated in writing the first draft of the manuscript (KMM, SD, GDCB). Revised and finalized the manuscript, and approved the acknowledgement of their contributions (KMM, SD, GB, AB, JPC, JH, LL, MST, KAA, GDCB, LT). All authors read and approved the final manuscript.

## Pre-publication history

The pre-publication history for this paper can be accessed here:

http://www.biomedcentral.com/1471-2431/14/161/prepub

## Supplementary Material

Additional file 1 CANPWR Project Office Staff, Coordinators, Investigators and Key Staff Project office staff (Population Health Research Institute, Hamilton Health Sciences and McMaster University, Hamilton, Canada). Click here for file
