# Supplementary material for: The CANadian Pediatric Weight Management Registry (CANPWR): Study protocol
Source: BMC Pediatr. 2014 Jun 23;14:161. doi: 10.1186/1471-2431-14-161 (PMC4082676; doi:10.1186/1471-2431-14-161)
Supplement: Additional file 1 — CANPWR Project Office Staff, Coordinators, Investigators and Key Staff Project office staff (Population Health Research Institute, Hamilton Health Sciences and McMaster University, Hamilton, Canada). [file 1471-2431-14-161-S1.doc]

**Appendix: CANPWR Project Office Staff, Coordinators, Investigators and Key Staff**

**Project office staff (Population Health Research Institute, Hamilton Health Sciences and McMaster University, Hamilton, Canada):** Coordination and Data Management: S Rangarajan (Project Manager); L Thabane, H Gerstein (External advisor ,Steering committee); B Feng (Statistician), J DeJesus, P Mackie, KM Morrison (Principal Investigator).

**BC CHILDREN’S HOSPITAL, Vancouver, British Columbia:** JP Chanoine*, J Kwan; **CHILDREN’S HOSPITAL OF EASTERN ONTARIO, Ottawa, Ontario:** A Bucholz*, MS Tremblay, S Hadjiyannakis, C Mohipp; **CHU SAINTE JUSTINE’S CHILDREN’S HOSPITAL, Montreal, Quebec:** AM LaBerge*, M Lambert, N Gaulin-Marion ; **MCMASTER CHILDREN’S HOSPITAL, Hamilton, Ontario:** KM Morrison*, M Jakymyshyn; **MONTREAL CHILDREN’S HOSPITAL, Montreal, Quebec:** L Legault*, C Farnesi; **NORTH YORK GENERAL HOSPITAL, Toronto, Ontario:** G Berall*, N Sutherland; **STOLLERY CHILDREN’S HOSPITAL, Edmonton, Alberta:** G Ball*, KA Ambler, S Damanhoury, M Yee; **THE HOSPITAL FOR SICK CHILDREN, Toronto, Ontario:** J Hamilton*, M Jamil.

*denotes Site Principal Investigator
